# Obturator hernia presenting with intestinal obstruction: A case study and CT imaging insights

**DOI:** 10.1016/j.radcr.2025.08.013

**Published:** 2025-08-28

**Authors:** Carlos Eduardo Lucio, Juan Camilo Ricardo, Lina María Acosta, Santiago Saavedra, María Carolina Pérez

**Affiliations:** a School of Medicine and Health Sciences, Universidad del Rosario, St. 12c #6-25, Bogotá, Colombia; b Department of Radiology and Diagnostic Imaging, Fundación Cardioinfantil-Instituto de Cardiología, St. 163a #13B-60, Bogotá, Colombia

**Keywords:** Abdominal hernia, Obturator hernia, Intestinal obstruction, Computed tomography, Acute abdomen

## Abstract

Obturator hernia is a rare but clinically significant cause of intestinal obstruction, particularly in elderly, thin women due to their anatomical predisposition. We present the case of a 79-year-old female with a history of hypertension and hip arthroplasty who developed acute abdominal pain, vomiting, and absence of flatus and bowel movements. Imaging with contrast-enhanced computed tomography (CT) revealed a right obturator hernia containing a small bowel loop, causing intestinal obstruction without signs of ischemia. The patient underwent urgent intraperitoneal obturator herniorrhaphy, during which an incarcerated small bowel loop with congestive changes was identified and found to be viable, recovering fully after reduction. This case highlights the importance of including obturator hernia in the differential diagnosis of bowel obstruction in elderly patients and demonstrates the pivotal role of CT imaging in achieving timely and accurate diagnosis.

## Introduction

Obturator hernias are an infrequent type of hernia that accounts for 0.07%-1% of all hernias [1]. It is defined by the protrusion of both intraperitoneal or extraperitoneal contents through the obturator canal [2]. There have been multiple factors associated with the development of these types of hernia including women, having a wider pelvis with an increased transverse diameter of the obturator canal, increased age, emaciation, multiparity, and those with increased intra-abdominal pressure such as patients with chronic obstructive pulmonary disease, chronic constipation or ascites [1,2].

## Case presentation

A 79 years old female with a history of systemic hypertension, chronic kidney disease with a right nephrostomy in place due to prior obstructive uropathy and left total hip replacement that required closed reduction the day prior due to luxation, with no personal or family history of gastrointestinal disease, hernias, or malignancy. She presented to the emergency department with a 20 hour history of abdominal pain associated with nausea, multiple emetic episodes which had resolved by the time of evaluation, absence of bowel movements and negative flatus. At the moment of admission, she had the following vital signs: Blood pressure of 164/85 mmHg, a heart rate of 99 bpm, a temperature of 36.0°C, and baseline O2 saturation of 96%. Generalized abdominal pain and distension were evidenced on physical examination with no other positive findings in relation to the clinical presentation.

Hemoglobin levels were 14.6 g/dL (reference range: 12-16), leukocyte count was elevated (12,750 cells/μL; reference range: 5000-10,000) with neutrophilia (11,030 cells/μL; reference range: 2000-7000), platelet count was 410.000 cells/μL (reference range: 150.000-450.000), creatinine 1.5 mg/dL (reference range: 0.6-1.1), ALT 52 U/L (reference: <55), AST 56 U/L (reference: <34), ALP 65 U/L (reference 30-130), total bilirubin 0.7 mg/dL (reference: <1.5) and Amylase (57 U/L reference: 28-100) and electrolyte levels were within normal range.

**Fig. 1:**
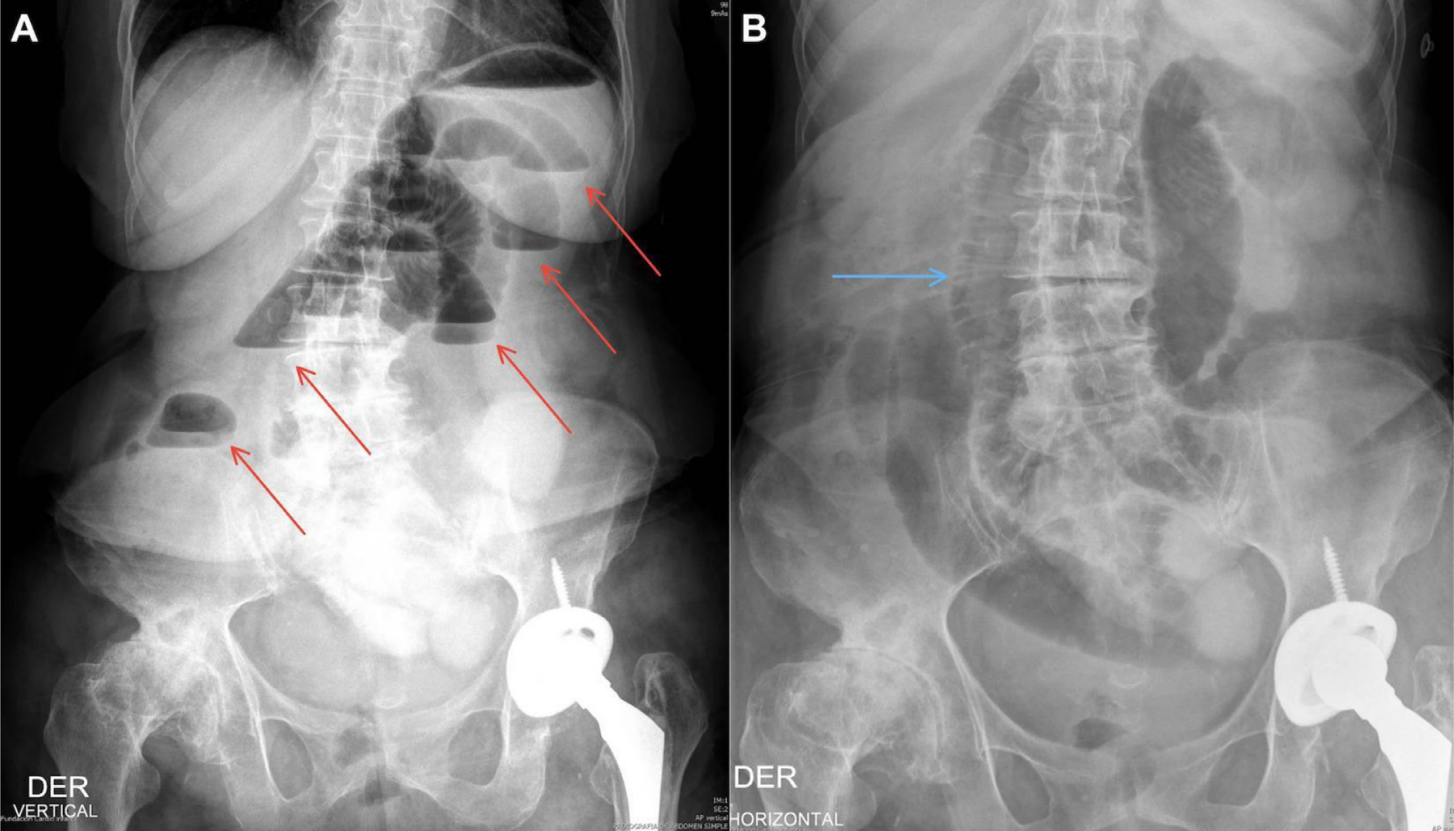
Abdomen radiographs in frontal projection in an upright position (A) and supine position (B). Distended small bowel loops with step-ladder air-fluid levels (red arrows) and thickened valvulae conniventes (blue arrow) are observed, findings indicative of small bowel obstruction. Additionally, severe degenerative changes are noted in the right hip joint, along with a total hip replacement on the left side.

**Fig. 2:**
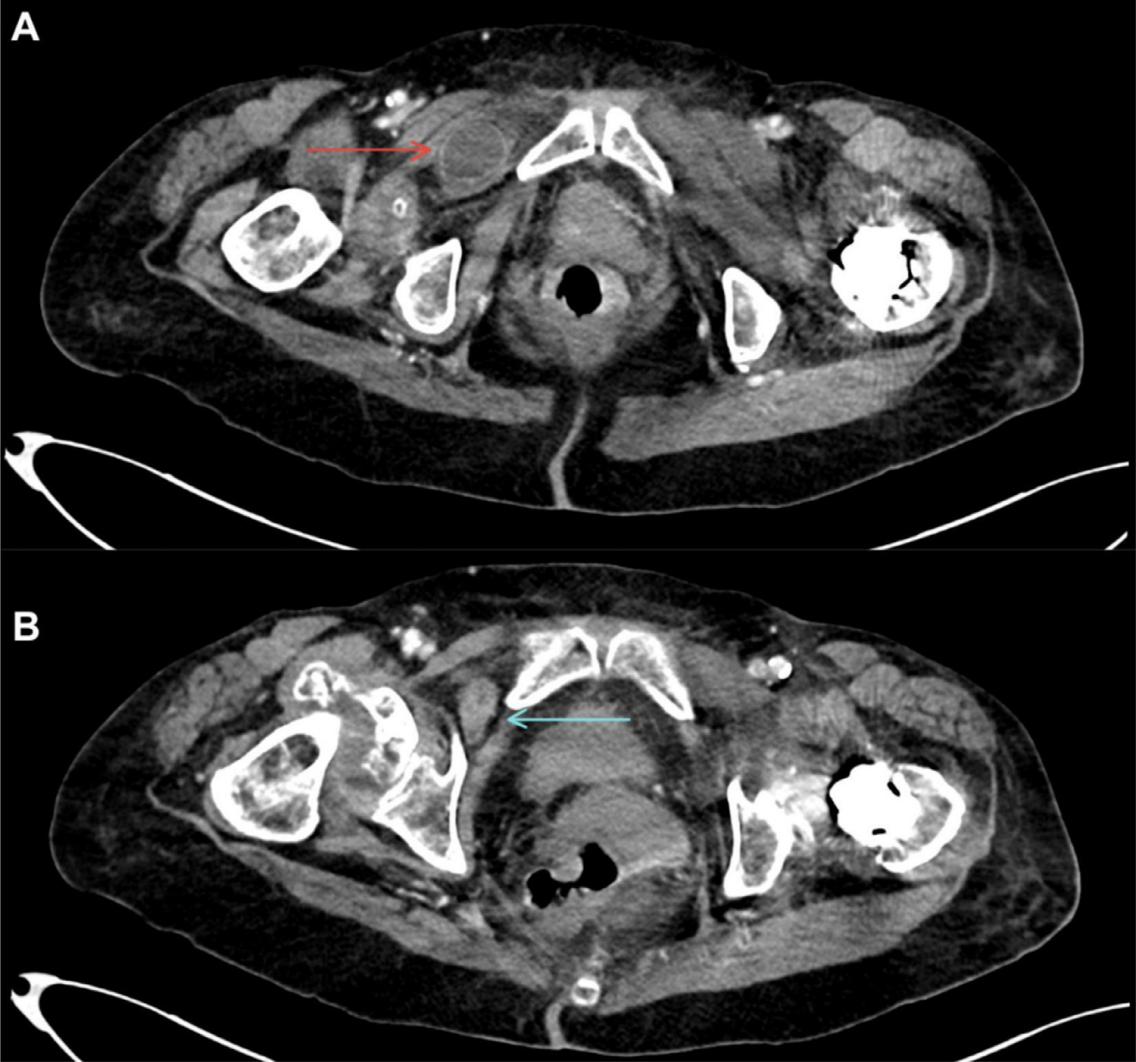
Contrast-enhanced abdominal computed tomography images in the portal venous phase, axial acquisition. (A) An ileal loop (red arrow) is identified between the fibers of the right external obturator muscle, deep to the right pectineus muscle. The loop shows no intraluminal contrast, has normal wall thickness, and demonstrates homogeneous enhancement. There is no evidence of hyperdense or loculated fluid within the hernia sac or adjacent mesenteric folds, nor is there intramural gas. Additionally, no portal venous gas or pneumoperitoneum is identified elsewhere in the study. (B) On a more cranial slice, the herniated loop is visualized passing through the right obturator foramen, appearing collapsed and creating a clear transition zone (blue arrow).

**Fig. 3:**
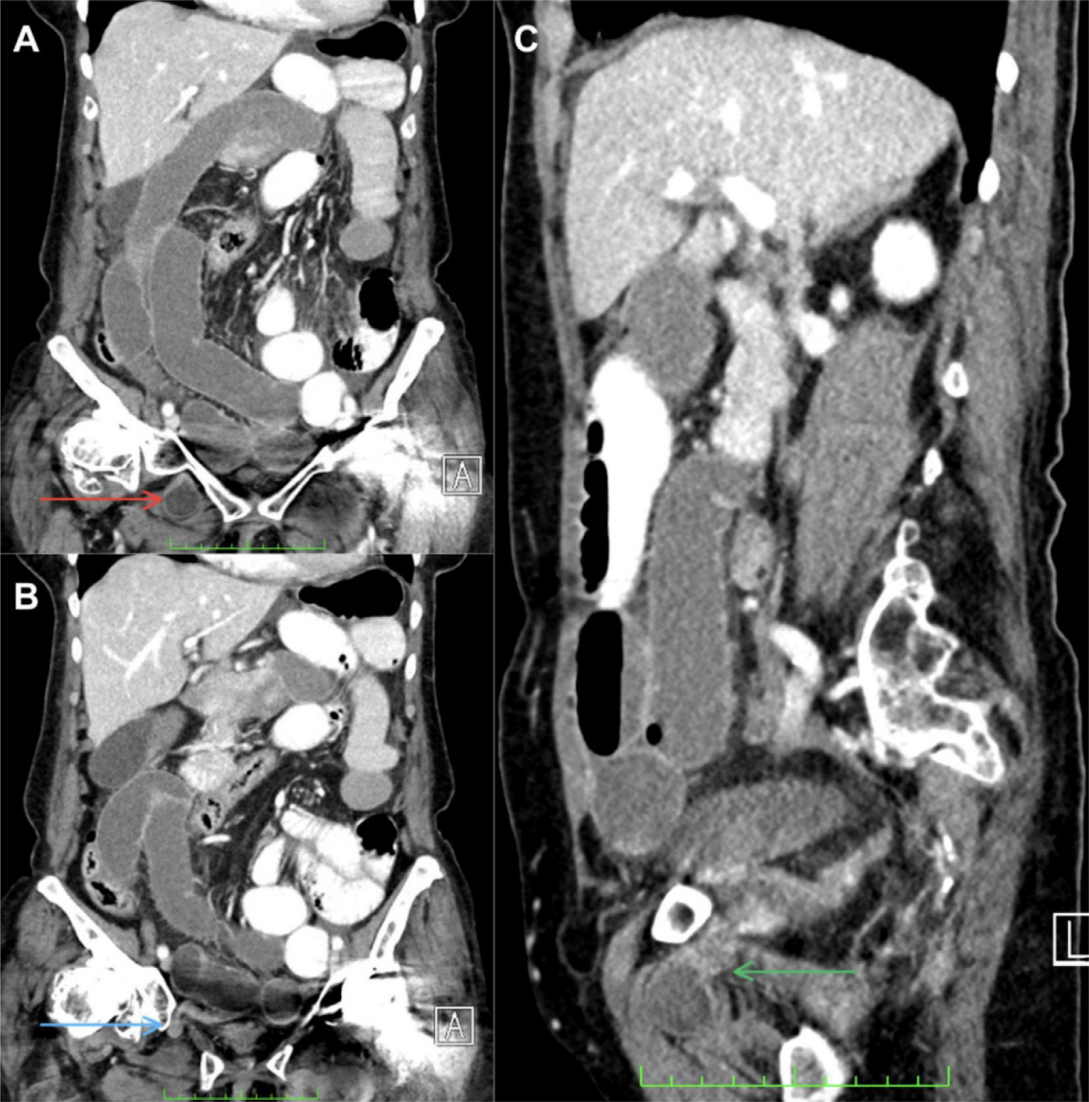
Contrast-enhanced abdominal computed tomography images in the portal venous phase, coronal reconstructions (A and B), and sagittal reconstruction (C). (A and B) An ileal loop is identified caudal to the right obturator foramen (red arrow), traversing this anatomical structure (blue and green arrows), where it appears collapsed, creating a transition zone. (C) Generalized dilation of small bowel loops is observed, along with the absence of intraluminal contrast in the intestinal loops within the right hemiabdomen, distal to the obturator hernia.

Conventional abdomen radiography revealed marked distension of intestinal loops, staggered air levels, vertebral degenerative changes, right hip arthrosis, and left hip replacement (Fig. 1). Only supine and upright abdominal radiographs were obtained. No lateral decubitus views were performed due to the patient’s recent orthopedic intervention and limited mobility. Given the patient’s stable condition, resolution of emetic episodes, the presence of distal rectal gas on the abdominal radiograph and absence of clinical findings suggestive of high-grade obstruction or ischemia, a low-grade small-bowel obstruction was initially suspected in the emergency department, and a contrast-enhanced CT scan of the abdomen and pelvis with oral and intravenous contrast was performed to better characterize the presence of a transition point. This evidenced a right obturator hernia with small intestinal loop content, an abdominal wall defect of 10 mm, and a hernia sac that had a longitudinal measure of 36 mm, an anteroposterior measure of 25 mm, and a transverse measure of 24 mm (Fig. 2). The involved small bowel loop demonstrated normal wall thickness and homogeneous enhancement following intravenous contrast, indicating preserved perfusion and absence of ischemia. No hyperdense or loculated fluid was seen within the hernia sac or adjacent mesenteric folds. There were no signs of pneumatosis intestinalis, portal venous gas, or pneumoperitoneum. There was evidence of generalized dilatation of the small intestinal loops, and the oral contrast was seen progressing through the proximal small bowel loops up to the transition point at the level of the obturator foramen, consistent with a mechanical obstruction with a closed-loop configuration. These findings supported the diagnosis of a non-complicated small bowel closed-loop obstruction secondary to an obturator hernia. The loops of the transverse, descending, and sigmoid colon were partially collapsed, along with scarce distal gas in the rectal ampulla (Fig. 3).

**Fig. 4:**
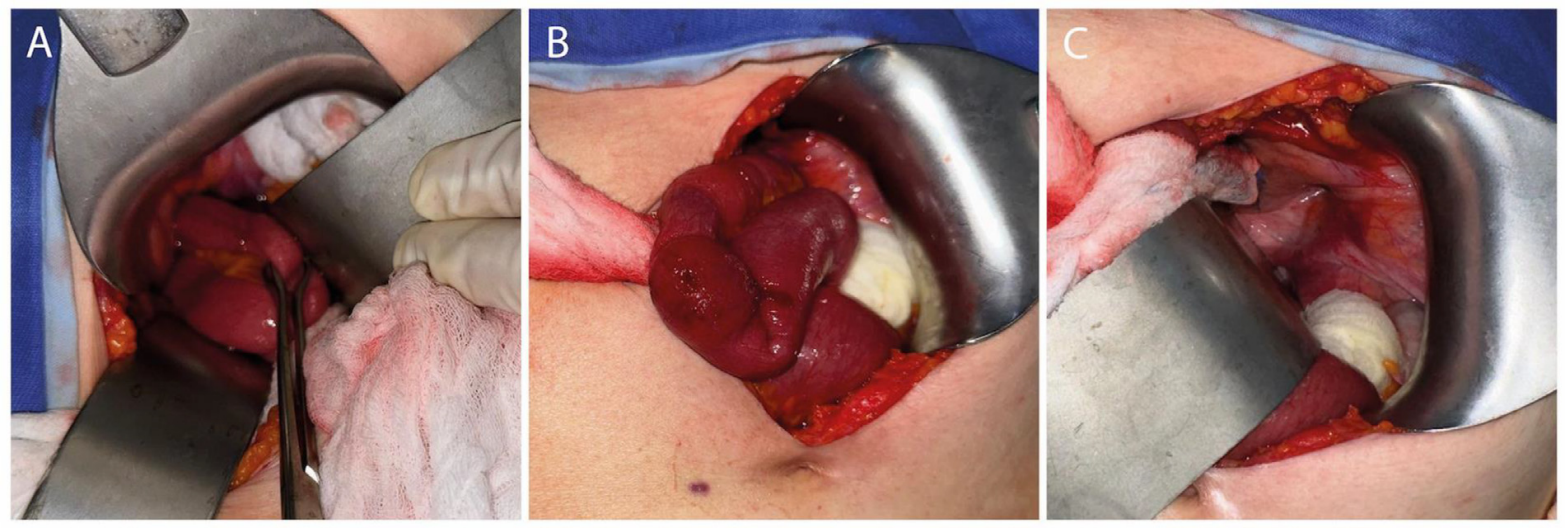
Intraoperative images showing: (A) a segment of the small bowel protruding through the right obturator foramen; (B) congestive appearance of a distal small bowel loop after hernia reduction; (C) the right obturator foramen after hernia reduction. CT, computed tomography.

Due to the image findings, the general surgery service was consulted. The patient underwent an open intraperitoneal obturator herniorrhaphy. Intraoperatively, an incarcerated right obturator hernia was identified, containing a segment of distal small bowel with congestive changes consistent with venous outflow impairment, which fully recovered after reduction. No resection was required, and a superficial erosion was noted in the affected loop (Fig. 4).

After four days of favorable and uneventful postoperative evolution, the patient was discharged from the hospital. Two weeks later, an outpatient follow-up showed no signs of postoperative complications, and the patient reported no pain, swelling, residual symptoms or other concerns at the surgical site. Normal activities were resumed without difficulty.

## Discussion

Obturator hernias are never externally visible nor palpable, so it is often undiagnosed or unsuspected and the surgical treatment is often delayed. Because of that, there is a significant increase in mortality and complications such as bowel gangrene leading to 25%-50% of patients with obturator hernias requiring bowel resection. [2,3].

The clinical presentation of patients with obturator hernias courses with non-specific signs and symptoms, most of these patients present signs of bowel obstruction, abdominal pain, vomiting, and nausea as was seen in our patient. Other manifestations may include fever or the presence of the Howship–Romberg sign, which is described as pain and dysesthesia in the groin, thigh, knee, and gluteal region on internal rotation of the hip, resulting from compression of the obturator nerve by the hernia. This sign is present in slightly more than half of patients (56.2%). [1,4].

Due to the difficulties in the clinical diagnosis of obturator hernias, image evaluation is key to detecting the presence of this kind of hernia. Certainly, the use of definite imaging in comparison to the use of plain abdominal radiographs for the diagnosis of obturator hernias results in less misdiagnosis and missed diagnosis, as well as a decrease in morbidity, mortality, and length of stay [1,5].

A CT scan of the abdomen and pelvis has proven to be the most sensitive and specific imaging technique for detecting obturator hernias. Studies have demonstrated an accuracy of 85%-100% for the diagnosis of obturator hernias [1,5,6,7].

Both computed tomography and magnetic resonance imaging easily diagnose obturator hernias, as these imaging techniques allow the identification of fluid, mesenteric fat, and/or intestinal loops traversing the obturator foramen. Additionally, they enable the visualization of associated complications, such as intestinal obstructions, signs of bowel ischemia, and perforation, among others [1,7,8]. In our case, the initial abdominal X-ray raised suspicion of intestinal obstruction, although the etiology was not identified. Consequently, a CT scan was performed, which revealed the passage of an ileal loop through the obturator foramen, associated with signs of intestinal obstruction.

Although an additional latero-lateral radiograph might have aided in visualizing a potential herniated bowel loop within the abdominal wall, this projection was not performed due to the patient’s limited mobility and recent hip manipulation. Given that the supine abdominal radiograph already showed findings suggestive of bowel obstruction, the patient was referred directly to CT for definitive evaluation.

The obturator hernia protrudes through the obturator foramen, an anatomical structure formed by the rami of the ischium and pubis. From there, it passes into the obturator canal, a narrow passage approximately 2-3 cm in length and about 1 cm in width, bordered by the obturator membrane and the pubic bone. This canal transmits the obturator nerve and vessels and serves as a potential space for herniation in predisposed individuals through the obturator internus and externus muscles [1]. Herniation through this confined space can entrap small bowel loops, particularly in elderly, emaciated females with pelvic widening and muscular atrophy, which further facilitates the descent of intra-abdominal contents [1].

Multiple operative approaches and surgical techniques for obturator hernia reduction and hernia defect closure have been reported in the literature. In terms of approaches, intraperitoneal or extraperitoneal are both viable options, the first one having the advantage of allowing to assess and treat possible bowel strangulation whereas the second one has a reduced rate of complications related to any open approach such as adhesion formation, postoperative ileus and risk of injury to other abdominal viscera [1].

In our patient, once the obturator hernia was identified on CT, surgical management was promptly indicated due to the potential risk of vascular compromise. An open intraperitoneal obturator herniorrhaphy was performed to allow direct assessment of bowel viability. Intraoperatively, a congested but viable segment of small bowel was identified within the hernia sac, with no signs of necrosis or need for resection. This surgical approach permitted immediate evaluation of bowel viability and ensured safe reduction of the incarcerated loop, an assessment that would not have been feasible with a purely extraperitoneal repair.

The real challenge in the differential diagnostic approach to obturator hernias lies in the etiologies that can cause intestinal obstruction. These causes are divided depending on whether they affect the small or large bowel. Within the former, we find that the most frequent etiologies are adhesions occupying 55%-75% of the cases, followed by hernias with 15%-25% and malignancies with 5%-10% of the cases. On the other hand, the most frequent causes of large bowel obstruction are cancer in 60% of cases, volvulus in 15%-20% of cases, and diverticular in 10% of cases [9]. The imaging findings of intestinal contents traversing the obturator foramen are specific to obturator hernias. Thus, once these were detected in our patient, the diagnosis was made.

## Conclusion

Obturator hernias, though rare, should be considered in elderly patients presenting with signs of bowel obstruction. Their nonspecific symptoms often delay diagnosis, increasing the risk of complications. This case highlights the essential role of computed tomography in accurately diagnosing obturator hernias and guiding timely surgical intervention. Early imaging and surgical management can significantly improve outcomes, as demonstrated in this patient’s favorable recovery.

## Authors’ contributions

All authors contributed equally to this manuscript’s conception, design, and writing. All authors have read and approved the final version of the manuscript.

## Ethical clearance

Not applicable.

## Patient consent

Written informed consent was obtained from the patient.
